# Composition of riparian litter input regulates organic matter decomposition: Implications for headwater stream functioning in a managed forest landscape

**DOI:** 10.1002/ece3.2726

**Published:** 2017-01-22

**Authors:** Johan Lidman, Micael Jonsson, Ryan M. Burrows, Mirco Bundschuh, Ryan A. Sponseller

**Affiliations:** ^1^ Department of Ecology and Environmental ScienceUmeå UniversityUmeåSweden; ^2^ Department of Forest Ecology and ManagementSwedish University of Agricultural SciencesUmeåSweden; ^3^ Australian Rivers InstituteGriffith UniversityNathan campusQldAustralia; ^4^ Department of Aquatic Science and AssessmentSwedish University of Agricultural SciencesUppsalaSweden

**Keywords:** boreal, introduced species, land use, litter quality, priming effect

## Abstract

Although the importance of stream condition for leaf litter decomposition has been extensively studied, little is known about how processing rates change in response to altered riparian vegetation community composition. We investigated patterns of plant litter input and decomposition across 20 boreal headwater streams that varied in proportions of riparian deciduous and coniferous trees. We measured a suite of in‐stream physical and chemical characteristics, as well as the amount and type of litter inputs from riparian vegetation, and related these to decomposition rates of native (alder, birch, and spruce) and introduced (lodgepole pine) litter species incubated in coarse‐ and fine‐mesh bags. Total litter inputs ranged more than fivefold among sites and increased with the proportion of deciduous vegetation in the riparian zone. In line with differences in initial litter quality, mean decomposition rate was highest for alder, followed by birch, spruce, and lodgepole pine (12, 55, and 68% lower rates, respectively). Further, these rates were greater in coarse‐mesh bags that allow colonization by macroinvertebrates. Variance in decomposition rate among sites for different species was best explained by different sets of environmental conditions, but litter‐input composition (i.e., quality) was overall highly important. On average, native litter decomposed faster in sites with higher‐quality litter input and (with the exception of spruce) higher concentrations of dissolved nutrients and open canopies. By contrast, lodgepole pine decomposed more rapidly in sites receiving lower‐quality litter inputs. Birch litter decomposition rate in coarse‐mesh bags was best predicted by the same environmental variables as in fine‐mesh bags, with additional positive influences of macroinvertebrate species richness. Hence, to facilitate energy turnover in boreal headwaters, forest management with focus on conifer production should aim at increasing the presence of native deciduous trees along streams, as they promote conditions that favor higher decomposition rates of terrestrial plant litter.

## 1 Introduction

Forested streams are often shaded by riparian vegetation, which constrains in‐stream primary production (Hill, Ryon, & Schilling, 1995) yet provides basal resources to aquatic food webs in the form of detritus (Naiman, Melillo, Lock, Ford, & Reice, 1987; Vannote, Minshall, Cummins, Sedell, & Cushing, 1980; Wallace, Eggert, Meyer, & Webster, 1997, 2015). In particular, the life cycles of many detritivorous aquatic insects are reliant on riparian plant litter inputs, timing their growth to seasonal peaks in litter fall (Richardson, 1991; Wallace, Eggert, Meyer, & Webster, 1999). In turn, the processing of litter by aquatic microbes and metazoans facilitates the transformation, cycling, and downstream transport of carbon (C) and nutrients in river networks (Rosemond et al., 2015; Wallace et al., 1991). As such, litter decomposition is of broad ecological and biogeochemical importance in streams and is increasingly targeted as a tool to assess the effects of environmental change in these ecosystems (e.g., Woodward et al., 2012).

Litter decomposition in streams is governed by both intrinsic and extrinsic factors that constrain rates of biological degradation (Tank, Rosi‐Marshall, Griffiths, Entrekin, & Stephen, 2010). First, different plant species produce litter that can differ greatly in terms of chemical composition, including the carbon‐to‐nitrogen (C:N) ratio and concentrations of secondary compounds (e.g., lignin, phenolics, tannins; Berg & Meentemeyer, 2002; Heal, Anderson, & Swift, 1997). These properties underpin differences in “litter quality” among species, leading to variation in rates of microbial colonization (Bärlocher, 1985; Graça, 2001) and growth (e.g., Gessner & Chauvet, 1994), feeding by invertebrate detritivores (Arsuffi & Suberkropp, 1989; Kiran, 1996), and overall rates of breakdown (Ostrofsky, 1997). In addition to these intrinsic constraints, decomposition may be further modified by stream temperature (Tank, Webster, & Benfield, 1993), concentrations of inorganic nutrients (Woodward et al., 2012), acidity (Simon, Simon, & Benfield, 2009), and the abundance and species richness of macroinvertebrate detritivores (Jonsson, Malmqvist, & Hoffsten, 2001).

Litter enters streams from riparian forests that can be notably diverse and, therefore, contribute a mix of litter species that differ in quality. Despite this diversity of inputs, most research has investigated how the quality of individual (or isolated) litter species influences decomposition rates. For studies that have tested how litter mixing regulates decomposition rates, results are equivocal: mixing high‐quality with low‐quality litter species may promote, retard, or show no effect on, overall decomposition rates (Hättenschwiler, Tiunov, & Scheu, 2005; Kominoski et al., 2007). At the same time, the direction and magnitude of litter‐mixing effects strongly depend on environmental context (Jonsson & Wardle, 2008; Leroy & Marks, 2006; Rosemond, Swan, Kominoski, & Dye, 2010). Further, results thus far suggest that litter‐mixing effects are less important in aquatic systems than in terrestrial soils (Gessner et al., 2010), probably due to a greater availability of organic and inorganic resources, and overall much faster decomposition rates, in water than in soils. As such, while research has shown that litter mixing may influence decomposition rates, the relative importance of these effects when compared to stream‐environmental conditions has not been well established (Tank et al., 2010).

Given the importance of litter quality for, and potential litter‐mixing effects on, litter decomposition rates, it is not surprising that some studies have found vegetation composition to regulate rates of local litter decomposition in streams (Jones & Swan, 2016; Kominoski, Marczak, & Richardson, 2011; Lecerf, Dobson, Dang, & Chauvet, 2005). Such effects may be attributed to (1) a local adaptation by microbial communities, resulting in locally produced litter being more readily decomposed than litter from outside (i.e., “home‐field advantage”; Gholz, Wedin, Smitherman, Harmon, & Parton, 2000; Hunt, Ingham, Coleman, Elliott, & Reid, 1988; Jackrel & Wootton, 2014) and/or (2) a “priming effect” where higher‐quality resources, by providing more easily accessible nutrients, increase microbial biomass, which in turn accelerates the turnover of lower‐quality resources (Guenet, Danger, Abbadie, & Lacroix, 2010; Kuzyakov, Friedel, & Stahr, 2000). Independent of the mechanism, these relationships have obvious implications for management of riparian zones, but the challenge is to assess the significance of riparian composition effects across heterogeneous landscapes, where a suite of other potentially important physical and chemical factors may also vary.

In Scandinavia, coniferous tree harvesting through clear‐cutting has been the dominant source of forest disturbance for over a century and continues to alter tree community composition in favor of commercially preferred coniferous species over deciduous counterparts (Esseen, Ehnström, Ericson, & Sjöberg, 1997; Laudon, Sponseller et al., 2011). It is not clear how this history of forest management influences the amount, composition, and turnover of in‐stream allochthonous resources. McKie and Malmqvist (2009) suggest that clear‐cutting along northern Swedish streams may accelerate local decomposition rates in the short term (3–5 years), potentially through inputs of early successional (deciduous) litter that attracts a greater abundance of macroinvertebrate detritivores. Yet, the effects of forestry on detrital inputs and processing as terrestrial succession ensues and deciduous trees are purposely removed through precommercial thinning, and, thus, gradually replaced by conifers, are not known. In addition, exotic plantations of lodgepole pine (*Pinus contorta*) now cover close to 600,000 ha, or approximately 3%, of the productive forest area in Sweden (Elfving, Ericsson, & Rosvall, 2001), including riparian zones of headwater streams.

Here, we ask how variation in the amount and composition of riparian litter inputs influences litter decomposition rates of three native and one exotic species in naturally vegetated boreal stream catchments. We investigated this interaction across 20 north‐Swedish catchments that encompass a gradient in forest regeneration ages (i.e., recently clear‐cut to 100+ years) and a corresponding shift from dominance by deciduous to coniferous species in the riparian zone (Jonsson et al., 2017). We measured microbial‐mediated decomposition rates of four litter species that differ in quality, and macroinvertebrate‐mediated decomposition of one deciduous litter species (i.e., birch), which was the species contributing the most to annual litter inputs. Besides in‐stream variables, such as water velocity, temperature, and nutrient concentrations, that are known to influence litter decomposition rates, we measured litter‐input quantity and composition, separated by seasons, to assess whether riparian vegetation litter production and community composition influence litter decomposition rates in boreal headwaters.

## 2 Materials and Methods

### 2.1 Study sites

**Figure 1:**
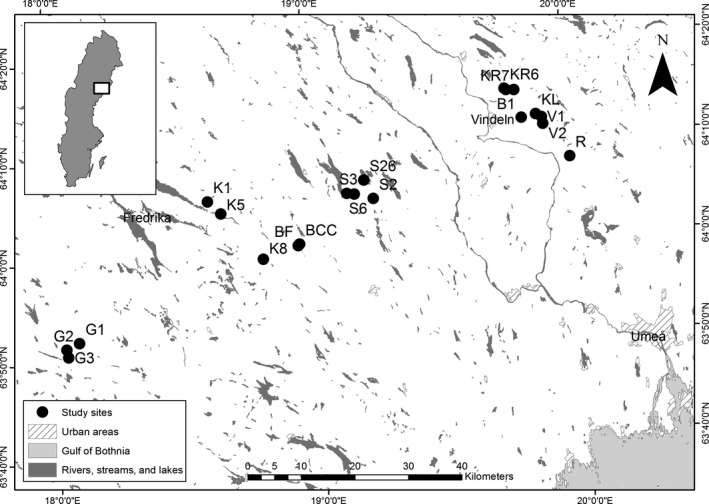
Locations of study sites in northern Sweden, including map coordinates. The inset shows the location of the study region in Sweden

The study was conducted in 20 first‐ and second‐order streams in the boreal landscape of northern Sweden (Figure 1). Our study sites were selected because they differ in riparian and catchment forest‐age structure and tree species composition, from young (recently clear‐cut) forests dominated by deciduous species to older forests dominated by conifers (Jonsson et al., 2017). Across our study sites, the most common tree species found in the riparian zones were Norway spruce (*Picea abies*), Scots pine (*Pinus sylvestris*), birch (*Betula* sp.), willow (*Salix* sp.), and alder (*Alnus* sp.). For each stream, a 100‐m reach dominated by riffles was selected as the study site. Along each of these reaches, we surveyed all riparian trees within 5 m of the stream bank to generate estimates of relative abundance for different broad‐leaved and coniferous species (Burrows et al., 2015).

### 2.2 Field sampling

The study was carried out from late May to early November, 2014. We monitored stream temperature continuously from mid‐August to early November, using HOBO^®^ pendant loggers attached to an iron bar approximately 5 cm above the streambed. As a proxy for local riparian forest‐age structure (i.e., time since clear‐cutting), light conditions (i.e., canopy openness) were estimated in August before leaf abscission, using a spherical densitometer. We measured stream width, depth (at both edges and in the middle), and velocity at three locations along each study reach. Due to seasonally fluctuating water levels, depth and stream velocity were measured three times during the study period (i.e., mid‐August, late September, and early November), to provide mean depth and velocity values for the entire period. Water samples were taken four times during the period (i.e., May, mid‐August, late September, and early November) for analysis of conductivity and pH, and to measure ambient concentrations of dissolved organic carbon (DOC), soluble reactive phosphorus (SRP), nitrate (${\text{NO}}_{3}^{-}$), ammonium (NH_4_^+^), and total nitrogen (TN). Water samples were filtered in the field with a 0.45‐μm nylon membrane filter (Sarstedt, Nümbrecht, Germany) and stored on ice until transferred to the laboratory refrigerator (4°C) or freezer (−25°C; for inorganic nutrients only) within 6 hr.

To quantify amount and type of litter input to each stream site, we placed three litter traps (2,000 cm^2^ in size) on the bank immediately adjacent to each reach in late May (Jonsson & Stenroth, 2016; Stenroth, Polvi, Fältström, & Jonsson, 2015). To capture differences in summer and autumn litter input, trapped litter was collected in late August, before deciduous leaf abscission, and again in late October once all deciduous litter had fallen and ice prevented further input of allochthonous resources to streams. As litter input is greater in autumn than in summer, and because input composition differs seasonally, we made the seasonal division to investigate whether characteristics of summer litter input could explain variation in autumn litter decomposition, or whether autumn litter‐input composition was the primary determinant of litter decomposition rates. The litter collected in the litter traps during the summer and autumn was sorted into six types (i.e., birch or alder leaves, spruce or pine needles, small woody debris [SWD], and “other”) and was oven‐dried at 60°C to a constant weight. The type “other” consisted mainly of grasses and herbs. The dried litter was then combusted at 550°C for 40 min to obtain ash‐free dry mass (AFDM) (Benfield, 1996).

### 2.3 Litter decomposition and litter input

To investigate leaf litter decomposition rates with respect to variation in riparian community composition, we selected litter from four different tree species. Three of these species (i.e., Norway spruce, birch, and alder) are native to northern Sweden, while lodgepole pine is introduced (Elfving et al., 2001). Dry needles of lodgepole pine and spruce were collected at separate locations in early June by shaking branches. Alder and birch leaves were also collected at single locations, but at abscission in early September 2013. All litter material was air‐dried indoors (~20°C) to a constant weight. The initial quality of litter used in the decomposition experiment was defined as the stoichiometric C:N ratio of dried litter for each species and was obtained from one bulk sample per species. To obtain this measure, C and N of the dried litter samples were by combustion converted to CO_2_ and N_2_, respectively. Then, mass spectrometric measurements (Flash EA 2000; Thermo Fisher Scientific, Bremen, Germany) yielded C and N quantities. All chemical analyses were performed by a certified laboratory at the Swedish University of Agricultural Science, Umeå, Sweden.

To measure microbial‐mediated leaf litter decomposition, 3.00 ± 0.05 g of litter material of each species was inserted in 15 × 15‐cm fine‐mesh (0.5 mm) bags, which excludes macroinvertebrate access. Another set of 12 × 17‐cm coarse‐mesh (5 mm) bags was used to measure leaf litter decomposition in the presence of both macroinvertebrates and microbes. Litter from all four species was used for the fine‐mesh bags, while only birch was used for the coarse‐mesh bags. Stalks on birch and alder leaves were removed before weighing and insertion into the bags, to increase accuracy in measures of consumable leaf litter mass (Jonsson et al., 2001).

At each site, five fine‐mesh litterbags of each litter species and five coarse‐mesh bags containing birch (i.e., 25 litterbags) were affixed at randomly selected locations to an anchored chain. Bags containing coniferous litter (i.e., spruce and lodgepole pine) were introduced in mid‐August, while bags containing deciduous litter were introduced in the end of September, to allow each species to reach similar stages of decomposition when collected. All litterbags were collected in mid‐November, rendering a total of 87–91 field days for coniferous litter and 53–56 field days for deciduous litter. The litterbags were brought to the laboratory and were frozen at −18°C, for later processing and analyses.

In the laboratory, litter removed from the bags was rinsed before being oven‐dried at 60°C for 48 hr. The dried litter was then combusted to obtain AFDM, following the procedure described above. Litter AFDM, used later in calculations of decomposition rates, was obtained by subtracting ash weight from dry mass. Following the same procedure, initial AFDM was obtained by combusting three 1.0‐g samples of each of the four litter species. To calculate decomposition rates for the four litter species, and for both fine‐ and coarse‐mesh bags, the decomposition constant (*k*) was calculated, using the negative exponential decay model (*k* = ln[*M*_t_/*M*_0_]/*t*), where *M*_t_ is the AFDM at time *t* and *M*_0_ is the initial AFDM.

### 2.4 Macroinvertebrates

We characterized the abundance, biomass, and species richness of macroinvertebrate detritivores residing within the coarse‐mesh litterbags (see Jonsson et al., 2001; for field method) to relate detritivore communities to litter decomposition rates at each site. While rinsing leaves in the laboratory, macroinvertebrates were removed and preserved in 70% ethanol before being sent to an expert taxonomist for identification to species or genera. Based on this identification, the macroinvertebrates were classified as belonging to the detritivore guild or not, according to a European database (Schmidt‐Kloiber & Hering, 2012). Detritivorous macroinvertebrates were then dried at 60°C for 48 hr to obtain an estimate of dry biomass per litterbag.

### 2.5 Water chemical analyses

Dissolved organic carbon and total nitrogen were analyzed using the combustion catalytic oxidation method on a Shimadzu TOCVCPH analyzer (Shimadzu, Duisburg, Germany). ${\text{NO}}_{3}^{-}$ (Method G‐384‐08 Rev. 2), ${\text{NH}}_{4}^{+}$ (Method G‐171‐96 Rev. 12), and SRP (Method G‐297‐03 Rev. 1) were analyzed using a SEAL Analytical AutoAnalyzer 3 (SEAL Analytical, Mequon, WI, U.S.A). Dissolved organic nitrogen (DON) was estimated as the difference between TN and total inorganic N (i.e., ${\text{NO}}_{3}^{-}$ + ${\text{NH}}_{4}^{+}$). From this, we calculated the DOC:DON ratio (mass) as a measure of “dissolved organic matter (DOM) character” that has been shown to be positively related to “humic‐like” compounds and inversely related to rates of microbial DOM degradation (e.g., Fellman, D'Amore, Hood, & Boone, 2008).

### 2.6 Statistical analyses

We used mixed‐effects models with site as a random factor to compare decomposition rates between fine‐ and coarse‐mesh litterbags (for birch only), among litter species (i.e., microbial decomposition), and to assess whether there were significant differences in input among different litter types and between seasons. This test was followed by pairwise comparisons using Tukey's HSD test. We used principal component analysis (PCA) to explore associations among seasonal (summer and autumn) patterns of litter inputs and physical and chemicals conditions. Moreover, to obtain litter‐input compositional variables (i.e., component 1 scores) that could be used in subsequent statistical analyses, we performed PCAs using only the summer or autumn senesced mass of different litter types. These compositional measures, which are distinct from absolute abundances of single litter species, were used as predictor variables for both microbial‐ (birch, alder, spruce, and lodgepole pine) and macroinvertebrate‐mediated (birch) litter decomposition rates. Hence, instead of only using the input of each litter type as a predictor variable, these component 1 scores allow us to test effects of the entire litter‐input community (i.e., mixture) on litter decomposition rates.

We used partial least squares (PLS) regressions to explore the predictors of litter decomposition from the suite of environmental variables. PLS regression relates two data matrices (i.e., predictor and dependent variables) to each other by a linear multivariate model and produces latent variables (PLS components) extracted from predictor variables that maximize the explained variance in the dependent variables. PLS regression is especially useful when predictor variables are correlated, and when there are more predictor variables than observations (Carrascal, Galvan, & Gordo, 2009). The evaluation of the PLS models was based on the level of variance explained (*R* ^2^), loadings of the independent variables, and the variable influence on projection (VIP). The independent variable loading describes the relative strength and direction of the relationship between independent and response variable. The VIP value summarizes the importance of each variable. In the models, the limit for a variable to be included in the final model was a VIP value at 0.7 (Eriksson et al., 2006). Neither PCA nor PLS regression assumes normally distributed data (Hulland, Ryan, & Rayner, 2010), but for the mixed‐effects models, variables were log‐transformed, if necessary, to meet the requirements of parametric statistical tests. All analyses were performed in R version 2.15.1 (R Core Team 2012) using the PLS package version 2.3‐0 for the PLS models (Mevik, Wehrens, & Liland, 2011).

## 3 Results

Among sites, mean stream width ranged from 39.7 to 185.0 cm, depth from 5.7 to 26.6 cm, velocity from 0.03 to 0.31 m/s, and water temperature from 3.8 to 4.9°C (see Table S1). Canopy openness was mostly ≤15%, with the exception of the recently harvested site (BCC; 87.4%). In terms of composition, spruce and birch were the most dominant riparian trees, and the dominant forms of conifer and broad‐leaved species, respectively (Table S1), and the relative abundance of these species was inversely correlated among sites (*r* = −.74, *p* < .001). DOC concentrations varied sixfold among sites (6.85–35.73 mg/L), the mass ratio of DOC:DON ranged from 48 to 80, pH varied from acidic (4.9) to circumneutral (6.4), SRP concentrations from 1.1 to 11.3 μg/L, and DIN concentrations were below 50 μg/L, except for at BCC (Table S2). Among sites, mean species richness, abundance, and biomass of the macroinvertebrate detritivores, based on individuals found in coarse‐mesh litterbags, ranged from 0.6 to 7.6 species, 6.0 to 113.2 individuals, and 1.2 to 16.3 mg DW, respectively (Table S3).

Estimates of total litter input from May to November ranged more than fivefold among sites, from 48.5 (±8.6) to 265.8 (±79.6) g AFDM /m^2^ (mean ± 1 SD). Total input increased across sites with the relative abundance of broad‐leaved trees in the riparian zone (*r* = .61, *p* = .004) and decreased with the abundance of spruce (*r* = −.65, *p* = .002). Similarly, the proportion of inputs composed of broad‐leaved material declined strongly among sites with spruce cover (*r* = −.80, *p* < .001). Seasonally, mean riparian plant litter input was five times lower in summer than in autumn, largely driven by significantly greater inputs of birch litter during autumn (*t* = −9.169, *p* < .001). During autumn, birch contributed the greatest amount of litter on average, followed by spruce, “other,” and alder, while pine and SWD contributed very little to the total input (Table 1). In contrast, summer litter input was quantitatively similar among litter types. Hence, the relative (%) contribution of different litter types to total amount deposited differed between seasons, with birch contributing significantly more in autumn, and SWD and “other” contributing more in summer (Table 1).

**Table 1:** Mean biomass for, and percentage of, different types of riparian litter input (±1 SE) during summer (May to mid‐August) and autumn (mid‐August to November), where alder, birch, and “other” are high‐quality litter, and pine, spruce, and small woody debris (SWD) are low‐quality litter

Litter type	Input (g AFDM/m^2^)		Input (%)	
	Summer	Autumn	Summer	Autumn
Alder	1.00 ± 0.60	11.05 ± 5.85	3.8 ± 1.8	7.5 ± 3.0
Birch	4.35 ± 0.80^b^	71.10 ± 11.25^a^	18.7 ± 3.8^b^	55.0 ± 4.9^a^
Other	6.85 ± 0.65	12.45 ± 2.00	29.1 ± 2.6^a^	10.5 ± 1.2^b^
Pine	2.00 ± 0.95	5.05 ± 2.00	6.6 ± 2.2	4.5 ± 1.4
Spruce	6.77 ± 1.45	18.05 ± 3.50	24.3 ± 4.5	19.3 ± 4.2
SWD	4.45 ± 0.90	3.75 ± 1.40	17.5 ± 2.4^a^	3.3 ± 1.3^b^

Different letters indicate significant differences between seasons at *p* = .05.

**Figure 2:**
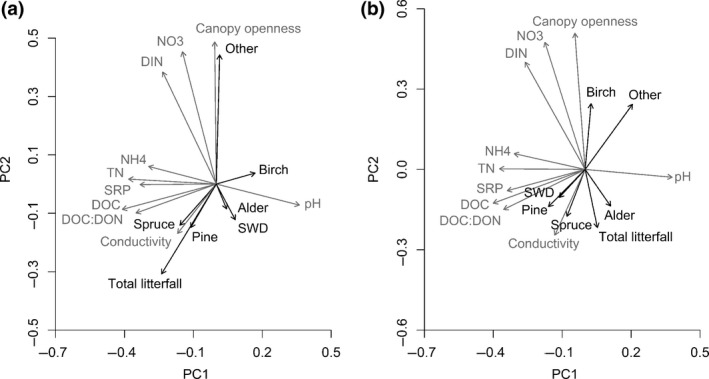
Results from principal component analyses (PCAs) showing associations among physical and water chemistry variables (gray arrows), and different types of riparian litter input (black arrows) in (a) summer and (b) autumn. Variance explained by PC1 and PC2, respectively, was 30.2% and 22.0% in summer and 26.7% and 18.1% in autumn

Patterns among environmental characteristics and litter types were consistent between seasons (Figure 2). More specifically, in both seasons, there was a positive association between spruce and pine litter, which was negatively associated with litter of birch and “other” (Figure 2). Moreover, spruce, pine, alder, and total litter input were negatively associated with canopy openness, and to concentrations of ${\text{NO}}_{3}^{-}$ and DIN, while input of “other” litter was positively related to these environmental conditions. Further, in both summer and autumn, input of low‐quality litter (i.e., SWD, pine, and to some extent spruce) was positively associated with the DOC:DON ratio. Total litter input was most strongly associated with spruce, pine, and “other” litter input in summer (Figure 2a), and more related to alder litter input in autumn (Figure 2b). PC1 in summer was positively associated with birch (Figure 2a), while PC1 in autumn was positively associated with a mixture of high‐quality litter types (i.e., birch, “other,” and alder; Figure 2b). In both seasons, PC2 was negatively associated with spruce and pine (Figure 2a,b).

**Figure 3:**
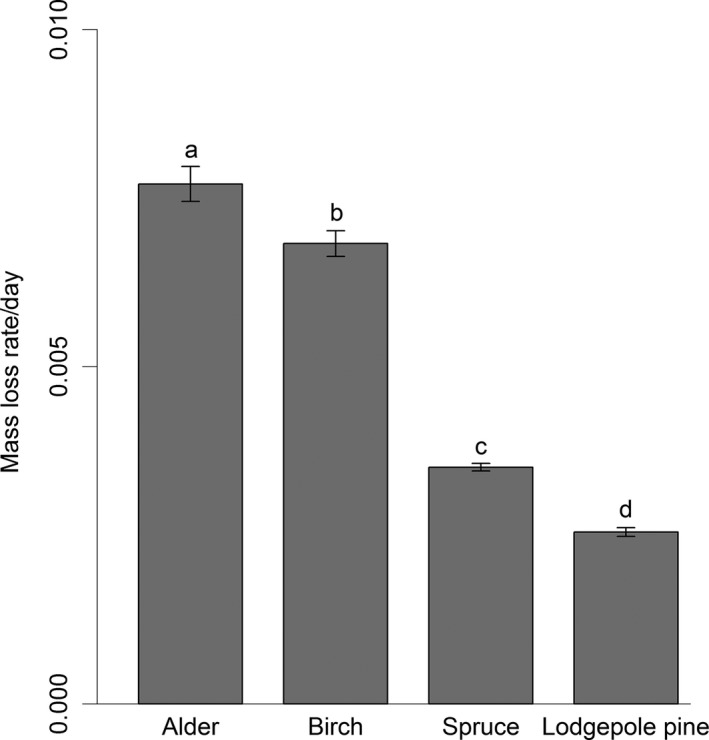
Litter mass loss rates in fine‐mesh litterbags (i.e., microbial decomposition) for alder, birch, spruce, and lodgepole pine (*n* = 20). Different small letters indicate significant differences at *p* = .05. Error bars represent ±1 SE

Mass loss rates (i.e., *k*) in the fine‐mesh litterbags differed significantly among all litter species (Tukey contrasts; *p* < .001 for all contrasts among species), with alder showing the highest *k* (mean = 0.0077, interquartile range: 0.0072–0.0083), followed by birch (mean = 0.0068, interquartile range: 0.0064–0.0072), spruce (mean = 0.0035, interquartile range:0.0033‐0.0037), and lodgepole pine (mean = 0.0025, interquartile range: 0.0024–0.0027; Figure 3). These decay constants reflect a total mass loss of 33% and 30% for alder and birch, respectively, over 53–56 days, which includes soluble materials leached at start of the incubation. By comparison, spruce and lodgepole pine lost only 27% and 20% of the original mass, respectively, over the longer (87–91 day) period of deployment. Overall, these differences in decomposition across species corresponded to similar differences in the C:N ratio, which was 16.6, 36.2, 45.1, and 51.3 for alder, birch, spruce, and lodgepole pine, respectively. Finally, *k* of birch litter was approximately 50% lower in fine‐mesh (mean = 0.0068, interquartile range: 0.0057–0.0079) than in coarse‐mesh (mean = 0.0144, interquartile range: 0.0133–0.0155) litterbags (*df *= 179, *t *= −23.8, *p* < .001), which on average lost 52% of the initial mass over 53–56 days (range of mass loss for coarse mesh: 39–70%).

**Figure 4:**
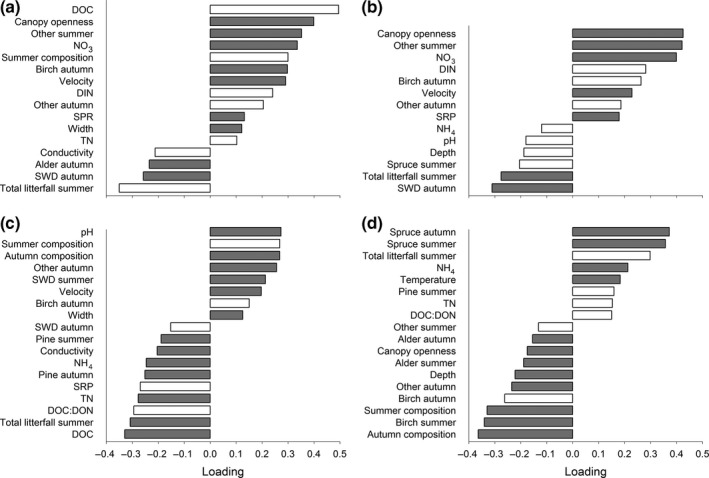
Results from PLS regression on litter mass loss in fine‐mesh litterbags for (a) alder, (b) birch, (c) spruce, and (d) lodgepole pine. Variance explained was 54.8%, 79.0%, 70.5%, and 60.5% (two components) for alder, birch, spruce, and lodgepole pine, respectively. Predictor variables with a VIP >0.7 are presented, and gray color indicates a VIP > 1.0

Partial least squares regression analyses on *k* from fine‐mesh bags ranked the importance of predictor variables differently among the four species, but for all species, there were several common variables that, through either positive or negative associations, best explained variation in *k* (Figure 4). Overall, with the exception of alder, litter‐input composition better explained *k* than did in‐stream physicochemical factors, such as water chemistry, temperature, and water velocity. For alder, the greatest *k* was found where DOC and canopy openness were high, and the lowest with high total litter input in summer (i.e., relatively high amounts of spruce and pine needle input) and autumn input of SWD (Figure 4a). In a similar way, *k* for birch litter was greatest in streams with high canopy openness, and the lowest with high inputs of the same types of poor‐quality litter as for alder (Figure 4b). However, in addition, birch *k* was positively associated with high‐quality litter input (i.e., “other” in summer and birch in autumn), and with nitrogen concentrations. Also *k* of spruce litter was positively associated with high‐quality litter input, that is, birch litter input (PC1, summer composition) and a mixture of birch, “other,” and alder (PC1, autumn composition), and negatively related to input of low‐quality litter (i.e., total summer input and pine input during autumn). However, in contrast to alder and birch, spruce litter showed higher *k* in streams with high pH and low DOC concentrations, and canopy openness did not show a significant association (Figure 4c).

**Figure 5:**
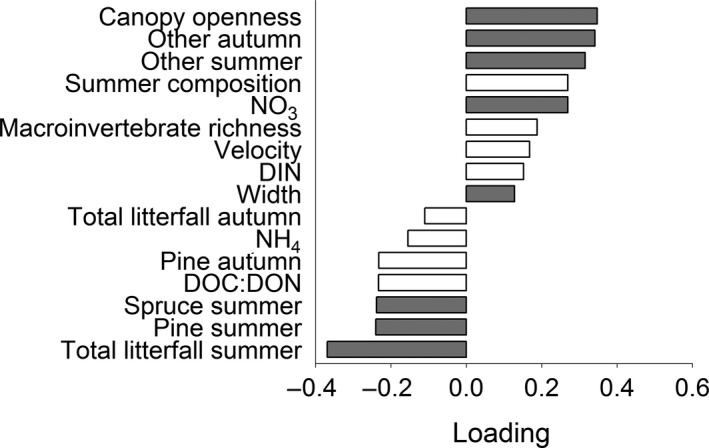
Results from PLS on birch litter mass loss in coarse‐mesh litterbags. Variance explained was 60.7% (two components). Predictor variables with a VIP > 0.7 are presented, and gray color indicates a VIP > 1.0

While the environmental factors that best predicted *k* were similar among the native species (Figure 4a–c), *k* of the introduced lodgepole pine was predicted by a different set of environmental conditions (Figure 4d). The most important factor associated with *k* of lodgepole pine was the quality of litter input, but *k* was greater in streams with high inputs of low‐quality litter (e.g., spruce in both summer and autumn), and negatively associated with high‐quality litter (i.e., summer and autumn composition, and birch in both summer and autumn; Figure 4d). Further, *k* of lodgepole pine litter showed a positive, albeit weak, relationship with DOC:DON (Figure 4d), while the opposite was found for mass loss rates of spruce litter (Figure 4c) and birch litter in coarse‐mesh bags (Figure 5), and ${\text{NH}}_{4}^{+}$ concentration was positively related only to lodgepole pine mass loss rates.

Birch *k* in the coarse‐mesh bags (Figure 5) was best explained by similar environmental variables as for the fine‐mesh bags (Figure 4b); that is, rates were highest in sites with high canopy openness, high‐quality litter input, and ${\text{NO}}_{3}^{-}$ concentration, and lowest in sites with low‐quality litter input. Macroinvertebrate detritivores also played a role, with greater mass loss in streams with greater detritivore species richness (Figure 5).

## 4 Discussion

Headwater streams across Fennoscandia are strongly influenced by management decisions that govern the composition and age structure of forests across vast areas (Esseen et al., 1997). Our results illustrate how these decisions drive variation in both the inputs and processing of terrestrial plant litter among headwater streams. Across this region, streams with riparian zones dominated by mature spruce forests received poorer‐quality litter at low amounts, with input rates that are comparable to annual estimates reported elsewhere at similar latitudes (ca. 30–60 g/m^2^; Benfield, 1997). By contrast, younger‐aged riparian stands dominated by broad‐leaved species contributed higher‐quality litter of considerably greater amounts (>250 g/m^2^). Between these extremes, the gradual replacement of deciduous trees by coniferous species resulted in predictable changes in the amount, quality, and timing of litter inputs. In this way, both the short‐term effects of forest management (i.e., clear‐cutting; McKie & Malmqvist, 2009) and the longer‐term outcome of successional change in the riparian zone (e.g., Hoover, Pinto, & Richardson, 2011) shape the broad‐scale patterns of litter supply to these headwaters.

This spatial variation in litter inputs was, in turn, related to differences in rates of decomposition across streams, and this was detectable despite the low variation in litter decomposition rates within litter species. Specifically, our results suggest that the input of high‐quality plant litter from the riparian zone may accelerate the decomposition of native litter species in boreal headwaters (cf. Frossard, Gerull, Mutz, & Gessner, 2013). Although we are not certain of the mechanisms involved, the composition (i.e., mixing) of the riparian plant community, together with in‐stream physicochemical variables, was clearly related to the variation in breakdown observed among sites. Moreover, the contrasting associations between litter‐input composition and decomposition rates for spruce and lodgepole pine indicate that such influences of litter mixing are context dependent (Jonsson & Wardle, 2008; Leroy & Marks, 2006; Rosemond et al., 2010) and, in this case, influenced by the presence of native or introduced tree species. Boreal forestry practices that alter and homogenize riparian tree community composition, reduce the presence of deciduous tree species through precommercial thinning in favor of commercially preferred coniferous species, and/or involve the introduction of non‐native conifers may therefore impact headwater stream functioning by altering basal processes in these aquatic food webs.

In general, litter decomposition was the highest in sites receiving greater inputs of high‐quality litter. These results corroborate previous studies (e.g., Kominoski et al., 2011; Lecerf et al., 2005; McKie & Malmqvist 2009), which have found that deciduous (i.e., high‐quality) litter input promotes litter decomposition rates. However, while previous studies have found effects only when macroinvertebrates were allowed access to the litter, we found these effects also in the absence of macroinvertebrates. Further, our results raise the possibility of an indirect priming effect from the input of high‐quality riparian litter on in‐stream litter of similar or poorer quality. However, litter decomposition rates were also often positively associated with canopy openness, and this could be due to higher levels of incident light stimulating in‐stream primary production (Hill et al., 1995) that, via a priming effect, may stimulate decomposition of terrestrially derived plant litter (Danger et al., 2013). Nevertheless, given our study design, high canopy openness was confounded by high amounts of higher‐quality litter, as more open canopies are found at sites where high‐quality litter input (i.e., birch or “other”) dominates (Figure 2).

In addition to influences of high‐quality litter, we found that microbial‐mediated litter decomposition rates were lowest in sites with poor‐quality organic matter input. Previous research has also shown reduced decomposition rates in mixtures containing poor‐quality litter, but only in the presence of macroinvertebrate detritivores (e.g., Kominoski et al., 2011; Lecerf et al., 2005). We found no evidence of environmental variables influencing decomposition rates via impacts on the macroinvertebrate detritivore community. Instead, macroinvertebrate‐mediated (birch) litter decomposition was best explained by the same environmental factors as was birch litter decomposition in the fine‐mesh bags (Figures 4b and 5), albeit with additional positive influences from macroinvertebrate species richness (Jonsson et al., 2001). The most plausible explanation to these results is that high presence of low‐quality litter reduces microbial diversity and/or biomass at the reach scale and thus the microbial‐mediated decomposition of the litter we deployed (e.g., Rosemond et al., 2010). However, whether variation in litter quality retards or promotes litter decomposition probably also depends on other environmental conditions, such as nutrient availability (Jonsson & Wardle, 2008; Rosemond et al., 2010), that we were unable to disentangle in this study.

Despite the overall importance of litter‐input composition (i.e., quality), the set of environmental factors that best predicted rates of litter decomposition differed among species. For both alder and birch litter, decomposition rates tended to increase with N concentrations, a result consistent with patterns of N limitation widely observed in other studies of litter processing (e.g., Woodward et al., 2012), and more specifically found for heterotrophic biofilm activity in these same streams (Burrows et al., 2015). In contrast, spruce litter decomposition was negatively associated with N concentrations and with DOC and DOC:DON, which, together with pH (positive), were the chemical variables most strongly associated with decomposition of this species. This negative relationship with DOC:DON could be a consequence of microbes using higher‐quality C from the water column rather than from the low‐quality spruce litter (Pastor et al., 2014), resulting in reduced decomposition rates. However, given covariation between DOC and pH (Laudon, Berggren et al., 2011; Figure 2), it is hard to separate the potential effects of DOM quality from those stemming from acidity, which is well known to reduce microbial litter processing rates in streams (e.g., Simon et al., 2009). The positive relationship between pH and spruce litter decomposition rate is also interesting given that sites where coniferous trees dominate also tend to have lower soil pH (e.g., Finzi, Canham, & van Breemen, 1998). Hence, besides being decomposed slowly due to low‐quality tissue, decomposition of spruce in streams may be further slowed by the acidic conditions that this species helps generate.

Lodgepole pine litter decomposed the slowest and was influenced by different variables, or by the same variables but in the opposite direction, compared to decomposition of native litter (Figure 4a–d). One reason for its lower rates of decomposition is that lodgepole pine litter was the least nutritious for microbial communities, reflected by its high C:N ratio compared to the other species studied. Yet, the C:N ratio of lodgepole pine was only slightly higher than that for spruce litter, which is consistent with results suggesting that concentrations of secondary compounds, such as lignin, are only marginally different between these species (Berg, 2000). Still, despite this marginal difference, we found a clear distinction between lodgepole pine and spruce in which environmental conditions influenced litter decomposition. This result suggests that a distinct microbial community may colonize and breakdown lodgepole pine litter (sensu Jackrel & Wootton, 2014) and that these microbes are influenced by a different array of environmental variables than those on associated native litter. However, microbial communities that should be adapted to low‐quality litter (i.e., spruce) seemed more able to utilize lodgepole pine as a resource (Figure 4d). Such communities are likely not as abundant in streams where high‐quality vegetation dominates the input (Frossard et al., 2013), and this could explain the low lodgepole pine litter decomposition rates in sites with high‐quality litter input, and conversely, the positive relationship between processing rate and coniferous litter inputs. Hence, we cannot rule out that the low decomposition rate of lodgepole pine is a consequence of few microbial groups found in Swedish boreal headwaters being adapted to its litter (sensu Gundale et al., 2014; Jackrel & Wootton, 2014), rather than it merely resulting from the low quality of this litter.

In summary, our results show how forestry practices govern the overall amount and quality of litter entering boreal headwaters and further indicate that a reduced presence of higher‐quality riparian plant species may lead to lower rates of leaf litter decomposition. In addition to these patterns and relationships, we also show that the non‐native lodgepole pine decomposed on average 22–68% slower than native counterparts. Future studies should thus investigate the consequences of more widespread use of this tree species for the energy transfer to consumers (e.g., invertebrates, fish) in adjacent and downstream aquatic food webs in this region. Ultimately, by enhancing both litter supply and biological processing rates, management decisions that lead to greater cover by deciduous riparian trees have the potential to strongly influence the transfer of energy through stream food webs in the boreal region.

## Data Accessibility

Data used in the study are housed in the Krycklan Experimental Catchment database (http://www.slu.se/en/krycklan).

## Conflict of Interest

None declared.
