# Supplementary material for: Composition of riparian litter input regulates organic matter decomposition: Implications for headwater stream functioning in a managed forest landscape
Source: Ecol Evol. 2017 Jan 22;7(4):1068–77. doi: 10.1002/ece3.2726 (PMC5305996; doi:10.1002/ece3.2726)
Supplement: Supplementary file 1 [file ECE3-7-1068-s001.docx]

**Supporting information**

**Table S1** Physical characteristics, and riparian vegetation composition, for each study site and the mean (± 1 SD) across all sites.

| Site | Width (cm) | Canopy openness (%) | Depth (cm) | Velocity (m s^-1^) | Temperature (°C) | Riparian vegetation composition | |
| --- | --- | --- | --- | --- | --- | --- | --- |
|  |  |  |  |  |  | % Spruce | % Birch |
| B1 | 121.8 | 5.7 | 22.8 | 0.03 | 4.4 | 77.8 | 22.2 |
| BCC | 43.8 | 87.4 | 7.0 | 0.13 | 4.4 | 77.8 | 22.2 |
| BF | 101.7 | 9.9 | 13.4 | 0.31 | 4.4 | 58.0 | 42.0 |
| G1 | 81.7 | 22.1 | 11.8 | 0.13 | 3.8 | 12.9 | 76.2 |
| G2 | 83.7 | 6.2 | 9.4 | 0.18 | 4.9 | 84.1 | 11.1 |
| G3 | 73.3 | 15.4 | 20.8 | 0.11 | 4.0 | 58.6 | 1.7 |
| K1 | 39.7 | 11.5 | 8.3 | 0.28 | 4.2 | 1.9 | 87.7 |
| K5 | 65.3 | 16.6 | 10.1 | 0.07 | 4.7 | 52.3 | 21.5 |
| K8 | 67.5 | 1.5 | 10.1 | 0.23 | 4.4 | 30.9 | 20.9 |
| KL | 144.3 | 3.2 | 26.6 | 0.25 | 4.1 | 22.6 | 53.4 |
| KR1 | 59.0 | 3.6 | 6.2 | 0.10 | 4.3 | 80.6 | 19.4 |
| KR6 | 84.7 | 8.6 | 9.9 | 0.10 | 4.5 | 94.1 | 5.9 |
| KR7 | 85.2 | 5.2 | 6.9 | 0.29 | 4.3 | 88.2 | 11.8 |
| R | 139.2 | 8.9 | 16.5 | 0.23 | 4.0 | 43.4 | 38.2 |
| S2 | 40.7 | 1.4 | 9.4 | 0.07 | 4.4 | 60.9 | 21.7 |
| S3 | 50.3 | 1.8 | 12.1 | 0.10 | 3.9 | 27.7 | 49.3 |
| S6 | 73.8 | 3.8 | 5.7 | 0.05 | 4.2 | 16.7 | 21.2 |
| S26 | 56.2 | 9.4 | 5.8 | 0.07 | 3.9 | 71.8 | 23.1 |
| V1 | 185.0 | 15.3 | 14.3 | 0.23 | 4.0 | 39.3 | 16.1 |
| V2 | 104.2 | 5.5 | 20.3 | 0.30 | 4.0 | 20.0 | 42.4 |
| Mean | 85.1 ± 38.3 | 12.5 ± 18.6 | 12.4 ± 6.1 | 0.16 ± 0.09 | 4.2 ± 0.3 | 51.0 ± 28.3 | 30.4 ± 22.5 |

**Table S2** Water chemistry for each study site and the mean (± 1 SD) across all sites.

| Site | pH | Conductivity  (µS cm^-1^) | SRP  (µg L^-1^) | NH_4_^+^  (µg L^-1^) | NO_3_^-^  (µg L^-1^) | DIN  (µg L^-1^) | TN  (mg L^-1^) | DOC  (mg L^-1^) | DOC:DON |
| --- | --- | --- | --- | --- | --- | --- | --- | --- | --- |
| B1 | 5.6 | 27.5 | 3.2 | 27.5 | 12.1 | 39.6 | 0.33 | 19.7 | 67.5 |
| BCC | 5.1 | 20.9 | 6.0 | 12.8 | 60.0 | 72.8 | 0.41 | 18.9 | 55.9 |
| BF | 5.4 | 22.6 | 5.0 | 7.1 | 7.0 | 14.1 | 0.37 | 19.6 | 54.8 |
| G1 | 5.3 | 15.7 | 3.1 | 5.7 | 5.7 | 11.4 | 0.28 | 17.1 | 63.9 |
| G2 | 6.4 | 22.3 | 1.1 | 7.3 | 8.1 | 15.4 | 0.27 | 13.0 | 50.2 |
| G3 | 6.5 | 15.2 | 1.3 | 3.7 | 7.0 | 10.6 | 0.15 | 6.9 | 49.3 |
| K1 | 6.2 | 19.9 | 6.1 | 5.5 | 7.5 | 13.0 | 0.16 | 9.2 | 58.6 |
| K5 | 6.1 | 17.7 | 1.4 | 4.8 | 11.5 | 16.2 | 0.17 | 7.6 | 50.4 |
| K8 | 6.0 | 15.8 | 2.5 | 11.2 | 17.7 | 28.9 | 0.35 | 19.2 | 59.3 |
| KL | 5.4 | 24.2 | 2.4 | 8.1 | 8.4 | 16.5 | 0.41 | 23.6 | 59.9 |
| KR1 | 5.5 | 29.5 | 2.6 | 20.7 | 28.1 | 48.8 | 0.43 | 23.2 | 60.4 |
| KR6 | 5.6 | 19.4 | 3.5 | 16.5 | 16.8 | 33.3 | 0.35 | 18.1 | 57.7 |
| KR7 | 4.9 | 30.2 | 9.9 | 11.9 | 16.5 | 28.4 | 0.44 | 27.3 | 65.4 |
| R | 6.2 | 29.3 | 2.0 | 9.3 | 18.8 | 28.1 | 0.37 | 17.7 | 51.9 |
| S2 | 5.0 | 39.1 | 11.3 | 11.3 | 9.2 | 20.5 | 0.47 | 35.7 | 79.5 |
| S3 | 5.4 | 28.5 | 4.0 | 10.9 | 7.9 | 18.8 | 0.41 | 21.3 | 48.4 |
| S6 | 5.7 | 44.3 | 1.9 | 5.9 | 16.8 | 22.7 | 0.34 | 15.2 | 55.0 |
| S26 | 6.7 | 39.0 | 1.5 | 4.3 | 8.8 | 13.1 | 0.23 | 10.3 | 48.7 |
| V1 | 6.2 | 28.3 | 2.1 | 7.2 | 14.3 | 21.4 | 0.32 | 17.2 | 55.8 |
| V2 | 6.2 | 27.0 | 1.8 | 5.6 | 9.2 | 14.8 | 0.28 | 15.1 | 56.6 |
| Mean | 5.8 ± 0.5 | 25.8 ± 8.1 | 3.7 ± 2.9 | 9.9 ± 6.0 | 14.6 ± 12.1 | 24.4 ± 15.2 | 0.33 ± 0.09 | 17.8 ± 6.8 | 57.5 ± 7.6 |

**Table S3** Macroinvertebrate detritivore species richness, abundance, and biomass for each site (mean litterbag^-1^; *n* = 5) and the mean (± 1 SD) across all sites.

| Site | Species richness  (number of species) | Abundance  (number of individuals) | Biomass  (mg DW) |
| --- | --- | --- | --- |
| B1 | 2.2 | 63.4 | 4.2 |
| BCC | 3.0 | 113.2 | 13.1 |
| BF | 4.6 | 22.4 | 4.1 |
| G1 | 2.8 | 24.0 | 1.2 |
| G2 | 4.6 | 16.8 | 3.5 |
| G3 | 2.4 | 6.0 | 2.0 |
| K1 | 4.8 | 79.6 | 4.2 |
| K5 | 2.2 | 9.4 | 4.3 |
| K8 | 3.2 | 30.2 | 3.7 |
| KL | 5.6 | 47.0 | 7.4 |
| KR1 | 3.2 | 26.2 | 2.3 |
| KR6 | 2.8 | 55.8 | 5.4 |
| KR7 | 1.6 | 26.8 | 1.9 |
| R | 7.6 | 33.8 | 11.7 |
| S2 | 3.0 | 103.8 | 7.3 |
| S3 | 0.6 | 35.8 | 2.1 |
| S6 | 1.8 | 46.0 | 9.2 |
| S26 | 2.6 | 7.0 | 16.3 |
| V1 | 4.2 | 15.8 | 3.5 |
| V2 | 4.0 | 14.0 | 6.1 |
| Mean | 3.3 ± 1.6 | 38.9 ± 30.7 | 5.7 ± 4.1 |
